# Multiple intramedullary nailing of proximal phalangeal fractures of hand

**DOI:** 10.4103/0019-5413.39573

**Published:** 2008

**Authors:** Hemant Patankar, Fayaz W Meman

**Affiliations:** Department of Orthopedics, Rajawadi Municipal Hospital, Mumbai, India; 1 Department of Orthopedics, Habib Hospital, Mumbai, India

**Keywords:** Hand, multiple intramedullary nailing, proximal phalangeal fractures

## Abstract

**Background:**

Proximal phalangeal fractures are commonly encountered fractures in the hand. Majority of them are stable and can be treated by non-operative means. However, unstable fractures i.e. those with shortening, displacement, angulation, rotational deformity or segmental fractures need surgical intervention. This prospective study was undertaken to evaluate the functional outcome after surgical stabilization of these fractures with joint-sparing multiple intramedullary nailing technique.

**Materials and Methods:**

Thirty-five patients with 35 isolated unstable proximal phalangeal shaft fractures of hand were managed by surgical stabilization with multiple intramedullary nailing technique. Fractures of the thumb were excluded. All the patients were followed up for a minimum of six months. They were assessed radiologically and clinically. The clinical evaluation was based on two criteria. 1. total active range of motion for digital functional assessment as suggested by the American Society for Surgery of Hand and 2. grip strength.

**Results:**

All the patients showed radiological union at six weeks. The overall results were excellent in all the patients. Adventitious bursitis was observed at the point of insertion of nails in one patient.

**Conclusion:**

Joint-sparing multiple intramedullary nailing of unstable proximal phalangeal fractures of hand provides satisfactory results with good functional outcome and fewer complications.

## INTRODUCTION

Phalangeal fractures are commonly encountered fractures in hand.^1^ Majority of them are stable and can be treated non-operatively. However, unstable fractures i.e. those with shortening, displacement, angulation, rotational deformity or segmental fractures need surgical intervention.^2^,^3^ The list of techniques described for the treatment of these fractures is exhaustive. The main aim of operative fracture fixation is to establish fracture stability and facilitate early mobilization of the hand to achieve functional recovery as early as possible.^2^,^4^

Intramedullary fixation of long bones is an accepted technique for more than five decades now.^5^ The use of multiple intramedullary nailing for small long bones of the hand was first described by Foucher G.^6^ Subsequently, the same was suitably modified for proximal phalangeal fractures of the hand by Hwa *et al.*^2^ and Gonzalez *et al*.^7^,^8^

The flexible nail incorporates three-point fixation. The technique of insertion of wires mimics that of Ender and Simon Wiedner.^5^,^9^ This technique is simple, safe and joint-sparing. Although it doesn't provide rigid fixation, it does provide fracture stability for early rehabilitation and functional recovery. Also, it is a very important technique that can be used in noncompliant patients.^10^ This prospective study was undertaken to evaluate the functional outcome after surgical stabilization of these fractures with joint-sparing multiple intramedullary nailing technique.

## MATERIALS AND METHODS

Thirty-five patients with 35 isolated unstable closed proximal phalangeal shaft fractures of the hand were treated with this method at two centers by two surgeons between 2001 and 2004. Fractures of the thumbs were excluded from the study. The mean age of our cohort was 27.3 years (range 15-45 years) and the male to female ratio was 35:0. Majority of the fractures were due to industrial accidents, sports injuries and fall. We had included transverse (*n* = 14,40%), short oblique (*n* = 12,34.29%), long oblique (*n* = 7,20%) and segmental fractures (*n* = 2,5.71%) in our study.

### Technique

All cases were operated under brachial block. With patient in supine position and hand to be operated over side arm support, tourniquet is inflated after standard scrubbing, painting, draping and limb exsanguination.

**Figure 1:**
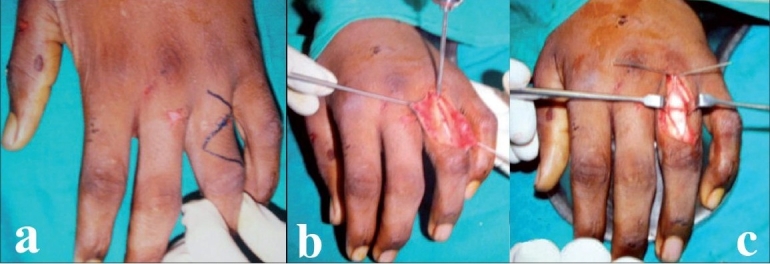
(a) Z incision over dorsum of proximal phalanx (b) Entry portal is metaphyseal and as far away from fracture as possible after splitting the extensor tendon (c) Fracture fixed with flexible nails (K-wires) inserted with bent tips in different directions

Z incision was made over the dorsum of proximal phalanx [Figure 1a]. Extensor tendon was split along midline to reach the bone. Entry portal site is metaphyseal and is selected as far away from fracture as possible [Figure 1b]. Proximal metaphyseal region was selected for antegrade nailing and distal metaphysis for retrograde insertion of nail.

**Figure 2:**
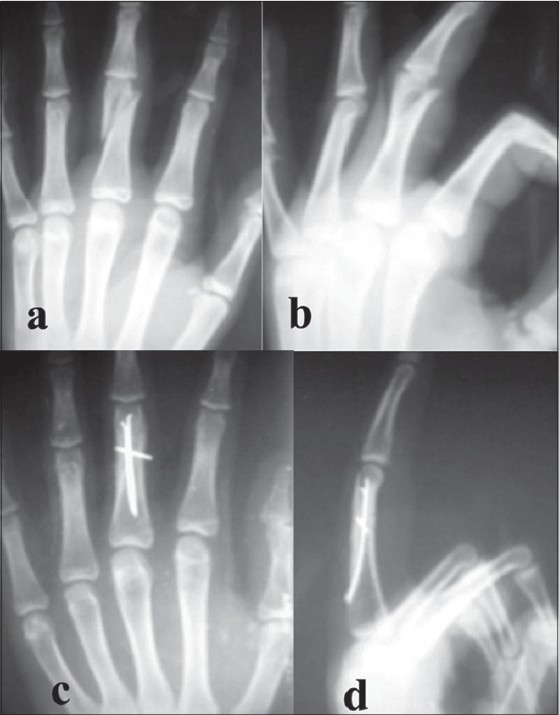
(a) Antero-posterior (AP) and (b) lateral view of right hand shows unstable long oblique fracture. AP and lateral X-ray (c and d) of right hand of the same patient shows additional interfragmentary K-wire for additional stability in long oblique fracture

A single hole was made with sharp 2.5-mm K-wire. This hole was drilled 45° towards the fracture site and location of intramedullary canal was confirmed with curved tipped 2.5-mm K-wire that acts as awl. Two to three, 0.8-mm blunt K-wires with their tips bent 20-30°, were used as nails, and were inserted into the medullary canal through the entry portal and advanced up to the fracture site. Fracture reduction was done and wires were further advanced till the end of the medullary canal (subchondral bone) [Figure 1(c)]. While advancing the wires were rotated so that their tips engage different quadrants from within, thus providing rotational stability. K-wires were cut as close to the bone as possible to avoid tethering of the extensor tendon. Extensor tendon was sutured with reverse knots and wound was closed. For unstable long oblique fracture, one or two interfragmentary K-wires were introduced for additional stability [Figure 2].

For the initial 15 cases, the above procedure was done by opening the fracture site. With experience the remaining cases could be done as a closed procedure. The fracture pattern and number of wires inserted are given in Table 1.

**Table 1:** Number of wires in each fracture pattern

No. of nails (K wires)	Fracture pattern			
	
	Transverse (*n* = 14)	Short oblique (*n* = 12)	Long oblique (*n* = 7)	Segmental (*n* = 2)
2	12	11	7	1
3	2	1	Nil	1
Additional intergragmentary K-wire(s)	Nil	Nil	All above fractures	Nil

Postoperatively, hand is placed in a plaster slab that maintains the metacarpophalangeal (MP) joint at 70-90° flexion while allowing full extension of interphalangeal (IP) joints. The operative time and duration of hospital stay were noted. Splint was maintained for three weeks, following which full extension and flexion of MP and IP joints was commenced under supervision. As all the fracture patterns operated were stable enough postoperatively, the rehabilitation program was the same for all fracture patterns. However, those fractures which were open reduced were observed closely for tendon adherence and scarring and merited vigorous physiotherapy to avoid the same. All the patients were followed up for a minimum of six months. All the patients were assessed clinically (at six weeks, 12 weeks, three months and six months) and radiologically (immediate postoperatively, after six weeks and after six months). The complications were also documented. The functional outcome was assessed at six months calculating Total Active range of Motion (TAM).^4^,^11^ This was done by adding the active flexion at metacarpophalangeal, proximal interphalangeal and distal interphalangeal joints, after subtracting the sum of extension deficit at these three joints. Recovery was calculated as percent-regained motion compared to normal range of digital motion (260°). According to this, patients with 85-100% of range of motion (ROM) were classified excellent; 70-84% as good; 50-69% as fair; and < 50% as poor.

## RESULTS

All the patients were followed up for a minimum of six months postoperatively. The average operative time was 30 min (range 15 min - 1 h) and average hospital stay was 1.3 days (range one to two days). With experience the operative time could be reduced to upto 15 min.

**Figure 3:**
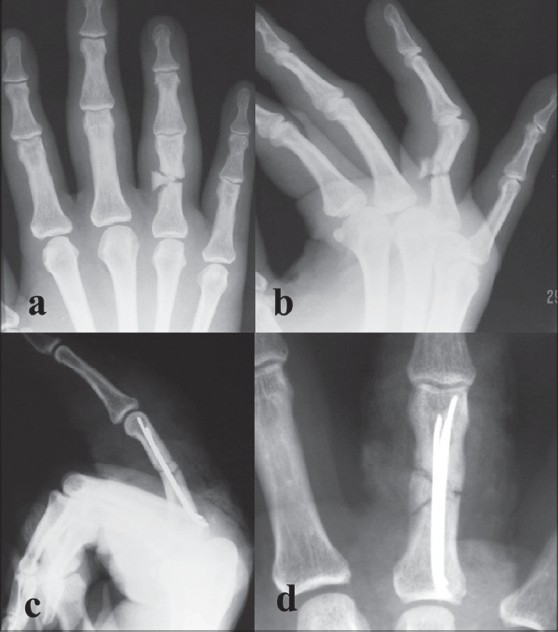
(a) AP view (b) lateral view of left hand shows unstable segmental proximal phalangeal fracture. Postoperative AP and lateral X-ray (c and d) of left hand of same patient shows segmental fracture fixed with multiple nails

The previous authors had not included long oblique fractures or segmental fractures in their studies with this technique,^2^,^8^ but we included them in our present study (long oblique *n* = 7, segmental *n* = 2) [Figures 2, 3]. Long oblique fractures required additional interfragmentary K-wire(s) for stability. There was no difference in the functional outcome of these fractures.

All the patients in our series had excellent TAM at six months. These are comparable to those obtained by Hwa *et al.*,^2^ Gonzalez *et al.*,^8^ for single digit involvement treated by this method. Our result was better as compared to other techniques for similar fractures.^12^–^15^ As our series included only isolated proximal phalangeal fractures of single digit, the results were better than those obtained by other authors who included multiple digits or metacarpal fractures in their series.^4^

Grip strength was achieved in all the patients at three months. The average grip strength was 32 kg (range 21-45 kg) on operated hand as compared to 33 kg (18 to 51 kg) on non-operated hand. This was similar to that obtained by Hwa *et al.*^2^ and Gonzalez *et al*.^8^

**Figure 4:**
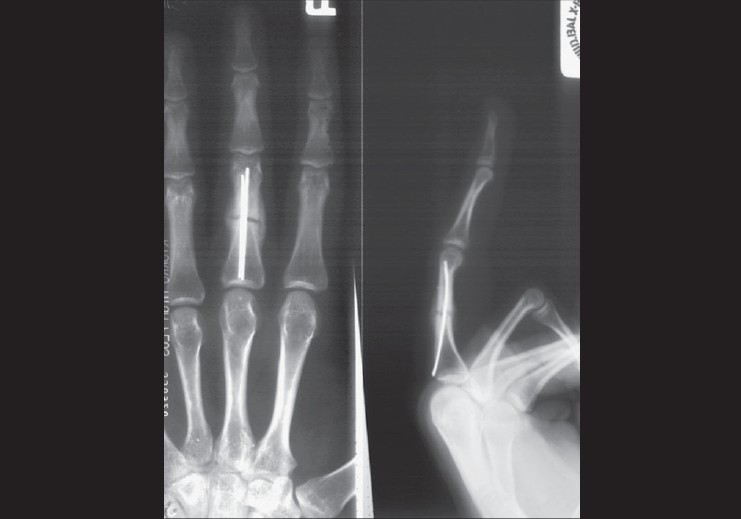
Anteroposterior and lateral X-ray views show radiological evidence of union in six weeks

Radiological evidence of fracture union was seen in all patients at six weeks [Figure 4]. There was no angulation, shortening, malunion or nonunion observed at six months. This was in contrast to previous authors^2^,^8^ who had reported variable degree of angulation in their series.

**Figure 5:**
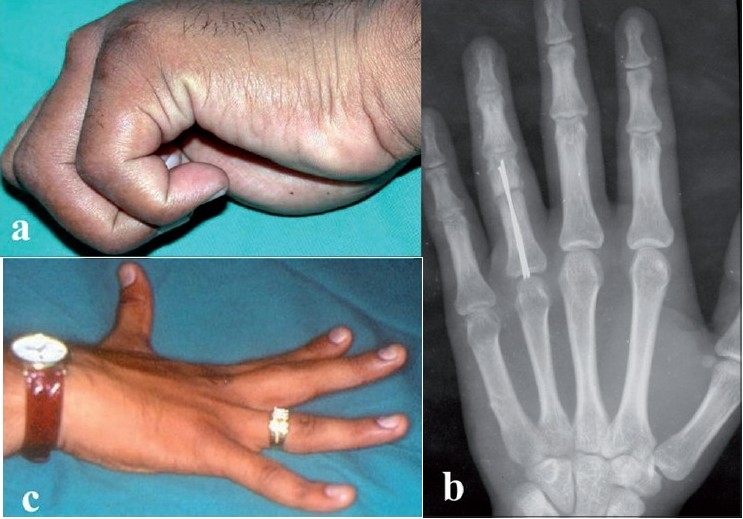
(a) Clinical photograph of adventitious bursitis at the site of entry portal in one patient (b) X-ray depecting of backing of nails causing adventitious bursitis. (c) Clinical photograph of 5° extension lag at proximal interphalangeal joint in one of the patients

One patient (3.33%) developed adventitious bursitis at entry portal site due to backing of nails [Figures 5 a, b]. This warranted removal of nails at five months. The fracture had united and patient had extension lag of 5° at proximal interphalangeal joint at six months.

Eleven patients (31.43%) had 5° extension lag at proximal interphalangeal joint at six months [Figure 5 c]. All these patients had undergone open reduction by extensor tendon splitting technique. There was no difference in functional outcome of individual digit fractures.

All the patients went back to their original work at an average of 3.5 months (range three to four months). None had to change their job.

## DISCUSSION

Although most of the proximal phalangeal fractures of the hand can be treated successfully by non-operative means, unstable fractures appear to benefit from operative fixation and stabilization.^2^ Studies on cadaver show that both flexion and extension forces of the hand decrease if shortening exceeds 3 mm or shaft angulation is more than 15°.^16^,^17^ The criteria for acceptable reduction in proximal phalangeal fractures is greater than 50% apposition, no rotational deformity, less than 15° angulation of fracture fragments in the antero-posterior (AP) plane and 10° in the medial lateral plane.^8^ The main indication of operative fixation is inability to achieve and maintain stable reduction.

Many fixation techniques are available including use of plates and screws, percutaneous pinning, cross K-wires and external fixations. Open reduction and internal fixation with plates and screws does provide rigid fixation, however, it needs periosteal stripping^8^,^14^,^15^ and tendon adherence about plate or screws has been described.^8^,^18^–^20^ Moreover it is expensive as compared to the K-wires which we used as flexible nails.

Percutaneous single pinning has the obvious disadvantage of not giving adequate rotational stability and also making it mandatory for implant removal.^21^ Further, keeping the pins out may cause serious pin tract infections.^12^,^22^,^23^ Use of cross K-wires have reported to create distraction of fracture fragments causing delayed union and nonunion.^24^

Intramedullary nailing of long bones of hand was introduced fairly recently.^6^,^25^ The concept is similar to the flexible nailing technique of Ender and Simon Weidner^9^ for the treatment of fractures of long bones. The main advantage of this technique is that it is simple, safe, joint-sparing and can be performed as a daycare procedure thus reducing the cost. It provides adequate stability due to three-point fixation by multiple nails thereby allowing early rehabilitation and functional recovery of hand. The avoidance of periosteal stripping is an added advantage in closed fixations. The only disadvantage of this technique is that it only gives stable internal fixation but not a rigid one and wires may bend with strong flexion forces. The previous authors had reported some angulation at fracture site at the end of their study.^2^,^8^ We tried to overcome this problem by inserting three nails wherever possible and increasing the immobilization time to three weeks post surgery rather than mobilizing immediately. The construct was stable enough and none of our patients had angulation at fracture site at the end of six months. None of the fractures lost reduction originally obtained in the operation theatre.^2^,^7^,^8^ Cutting the nails flush with the bone avoided soft tissue tethering and without the need for implant removal except in one case with adventitious bursitis due to backing of nails. Aggressive occupational therapy under supervision is necessary after three weeks to avoid scarring and tendon adherence.

In the present series we got 100% excellent results which was similar to Hwa *et al.*^2^ and Gonzalez *et al.*,^7^,^8^. Moreover, our complication rate was less (*n* = 1, 3.33%), probably due to strict patient selection with only closed isolated proximal phalangeal fracture included in our series. Use of other techniques for similar personality of fractures by various authors have yielded inferior results or higher complications.^4^,^12^,^13^,^18^,^21^

## CONCLUSION

This technique represents a safe and effective treatment option for unstable proximal phalangeal fractures of hand. It provides maximum functional recovery with minimal complication rate. This technique is not recommended for intraarticular fractures and those with extremely short fracture fragments i.e. too distal or too proximal fractures.
